# Tumor-treating fields (TTFields) induce immunogenic cell death resulting in enhanced antitumor efficacy when combined with anti-PD-1 therapy

**DOI:** 10.1007/s00262-020-02534-7

**Published:** 2020-03-06

**Authors:** Tali Voloshin, Noa Kaynan, Shiri Davidi, Yaara Porat, Anna Shteingauz, Rosa S. Schneiderman, Einav Zeevi, Mijal Munster, Roni Blat, Catherine Tempel Brami, Shay Cahal, Aviran Itzhaki, Moshe Giladi, Eilon D. Kirson, Uri Weinberg, Adrian Kinzel, Yoram Palti

**Affiliations:** 1 Novocure Ltd., Topaz Building, MATAM Center, 31905 Haifa, Israel; 2 Novocure GmbH, Munich, Germany

**Keywords:** Tumor-treating fields, Anti-PD-1, ER stress, Immunogenic cell death, Autophagy

## Abstract

Tumor-treating fields (TTFields) are alternating electric fields in a specific frequency range (100–300 kHz) delivered to the human body through transducer arrays. In this study, we evaluated whether TTFields-mediated cell death can elicit antitumoral immunity and hence would be effectively combined with anti-PD-1 therapy. We demonstrate that in TTFields-treated cancer cells, damage-associated molecular patterns including high-mobility group B1 and adenosine triphosphate are released and calreticulin is exposed on the cell surface. Moreover, we show that TTFields treatment promotes the engulfment of cancer cells by dendritic cells (DCs) and DCs maturation in vitro, as well as recruitment of immune cells in vivo. Additionally, our study demonstrates that the combination of TTFields with anti-PD-1 therapy results in a significant decline of tumor volume and increase in the percentage of tumor-infiltrating leukocytes in two tumor models. In orthotopic lung tumors, these infiltrating leukocytes, specifically macrophages and DCs, showed elevated expression of PD-L1. Compatibly, cytotoxic T-cells isolated from these tumors demonstrated increased production of IFN-γ. In colon cancer tumors, T-cells infiltration was significantly increased following long treatment duration with TTFields plus anti-PD-1. Collectively, our results suggest that TTFields therapy can induce anticancer immune response. Furthermore, we demonstrate robust efficacy of concomitant application of TTFields and anti-PD-1 therapy. These data suggest that integrating TTFields with anti-PD-1 therapy may further enhance antitumor immunity, hence achieve better tumor control.

**Electronic supplementary material:**

The online version of this article (10.1007/s00262-020-02534-7) contains supplementary material, which is available to authorized users.

## Introduction

The field of cancer immunotherapy has gone through tremendous development over the past decade. Despite the recent breakthroughs achieved by the use of immune checkpoint inhibitors, the majority of cancer patients still do not respond to anti-PD-1/PD-L1 therapy [1]. This could be attributed to a failure in accomplishing the early steps in the cancer-immunity cycle [2, 3]. The success of such drugs is thereby largely dependent on a strong, preexisting, endogenous immune response [4]. It was suggested that specific drug combinations may elicit earlier “upstream” steps, which could eventually drive the entire cycle into a successive antitumor immune response [3].

Tumor-treating fields (TTFields), an anticancerous treatment modality, are alternating electric fields with low intensity (1–3 V/cm) and intermediate frequency (100–300 kHz) that are delivered in a noninvasive manner [5]. TTFields therapy is clinically applied for the treatment of glioblastoma multiforme and malignant pleural mesothelioma. Previous studies have shown that TTFields impair the polymerization of microtubules and septin filaments, which are required during mitosis for proper chromosome segregation and cytokinesis [6, 7]. Correspondingly, TTFields application to dividing cells results in mitotic catastrophe leading to aneuploidy and subsequent cell death [6, 7].

Here, we investigated the potential of TTFields therapy to induce immunogenic cell death (ICD) and initiate adaptive immunity by providing inflammatory stimuli for DCs. We also evaluated the efficacy of concurrent application of TTFields and anti-PD-1 therapy in orthotropic murine Lewis lung carcinoma and heterotopic subcutaneous colon cancer models.

## Materials and methods

### Tumor cell lines

The cell lines used for this study were Lewis lung carcinoma (LLC-1), CT-26 murine colon carcinoma, HEPG2 (HB-8065) human hepatocellular carcinoma, H520 (HTB-182) human lung squamous cell carcinoma, and spontaneously transformed murine ovarian surface epithelial (MOSE-L).

### TTFields application in vitro

TTFields were applied for 24–72 h using the Inovitro™ system (Novocure, Israel) as previously described [5, 8]. Cells were treated with TTFields at intensity of 175 V/m (RMS) and frequencies of 150 kHz (LLC-1, H520, and HEPG2) and 200 kHz (CT-26, MOSE-L).

### Cell count

Inhibition of cell growth was analyzed by quantitatively determining cell count using iCyt EC800 flow cytometer (Sony Biotechnology). The relative number of cells at the end of treatment was expressed as a percentage of untreated control cells.

### Quantification of cell death

Cell death was assessed by double staining of cells with FITC-conjugated annexin V (MEBCYTO^©^ 4700 Apoptosis Kit; MBL^©^) and 7-Aminoactinomycin D (7-AAD; BioLegend^©^) as per manufacturer’s instructions.

### Analysis of calreticulin (CRT) exposure on the cell surface

Cells were incubated with rabbit anti-mouse calreticulin antibody (1:200; Abcam), followed by incubation with donkey anti-rabbit Alexa Fluor 488 conjugated antibody (1:250; Jackson ImmunoResearch).

### Detection of ATP levels in cells exposed to TTFields

Cells were loaded with 1 μM quinacrine (Sigma-Aldrich^©^). Cellular ATP levels were measured by quantification of the percentage of viable cells (7AAD-) that exhibited strong reduction in quinacrine staining (Quinacrine-/7AAD-). Alternatively, supernatants were used for quantification of ATP release using the ENLITEN^®^ ATP Assay System (Promega).

### Electron microscopy

Thin sections (70 nm) were coated with carbon and visualized using Zeiss Ultra-Plus FEG-SEM equipped with transmission electron detector, at acceleration voltage of 30 kV.

### Detection of HMGB1 release

Supernatants were collected and were used for quantification of HMGB1 by ELISA assay (IBL International GmbH).

### Immunofluorescence

Microtubule-associated protein 1 light chain 3 (LC3) detecting antibody (rabbit polyclonal, Novus) and Alexa Fluor 488-conjugated secondary antibody (Jackson ImmunoResearch) were used. Images were collected using LSM 700 laser scanning confocal system, attached to an upright motorized microscope with × 63/1.40 oil objective (ZeissAxio Imager Z2). For detection of T-cells, frozen sections (7 µm thick) were stained with anti-CD8 primary antibody (YTS169.4, abcam), followed by secondary Cy3-conjugated donkey anti-mouse antibody (Jackson ImmunoResearch). The whole slide image was collected using automatic slide scanner 250 Flash (3DHISTECH). For immunohistochemistry staining, lungs were embedded in paraffin and staining was performed on 4-µm sections using the Leica Bond max system (Leica Biosystems Newcastle Ltd, UK). Sections were dewaxed and pretreated with epitope retrieval solution (Leica Biosystems Newcastle Ltd, UK) followed by anti-CD45 antibody (1:600, ab10558 by Abcam).

### Immunoblotting

Anti-LC3 was purchased from Novus, phospho-eIF2α (Ser 51) and eIF2α detecting antibodies from Cell signaling, anti-vinculin from Sigma-Aldrich, anti-GAPDH from Santa-Cruz, followed by HRP-conjugated secondary antibody (Abcam) and a chemiluminescent substrate (Millipore). Densitometric analysis was performed with the Image Studio Lite 5.2 software (LI-COR).

### Efficacy of TTFields and anti-PD-1 combination therapy in animal models

For orthotopic lung cancer model, 10–12-week-old male C57Bl/6 mice were injected directly into the lungs with LLC-1 cells. For heterotopic subcutaneous colon cancer model, 0.5 × 10^6^ CT-26 cells in 200 µL PBS were subcutaneously injected to the upper right flank of 10-week-old female Balb/c mice. Mice received an I.P. injection of anti-PD-1 (RMP1-14; 250 μg) or Rat IgG2a (2A3; 250 μg) (Bioxcell). Tumor size was assessed with Vernier calipers using the formula width^2^ × length × 0.5.

For more details, see Figs. 5a, 6a, and S5.

### Tumor tissue processing

The tumors were prepared as single-cell suspensions using the gentleMACS™ dissociator and the tumor dissociation kit (Miltenyi Biotec GmbH, Gladbach, Germany).

### Isolation and activation of tumor infiltrating leukocytes (TILs)

TILs were isolated by dissociating tumor tissue as described above. Single-cell suspensions were filtered and separated by Mouse Pan T magnetic bead selection (Invitrogen). Isolated cells were then cultured in vitro in the presence of T-Activator CD3/CD28 magnetic beads (Invitrogen) for 24 h.

### Isolation and priming of DCs

For the generation of bone marrow-derived DCs (BMDCs), bone marrow cells were flushed from the femurs and tibias of 5–7-week-old C57BL/6 mice. For DC maturation assay, TTFields-treated cells were added at a ratio of 1:1 for 24 h. For phagocytosis assay, CellTracker™ Deep Red Dye (25 μM; Invitrogen) pre-stained cells were added at a ratio of 1:1 for 2 h.

### Peritoneal inflammation

10–12-week-old male C57Bl/6 mice were injected to the peritoneal cavity with 200 µL PBS or 200 µL PBS containing 2 × 10^6^ control, TTFields-treated cells, and freeze/thawed (F/T)-treated cells. Following 48 h, mice were euthanized. Infiltration of immune cells (CD45+) was evaluated using flow cytometry.

### Flow cytometry

Fluorochrome-conjugated anti-mouse CD45 (clone 30-F11), CD8a (clone53-6.7), F4/80 (clone BM8), PD-L1 (clone 10F.9G2), CD4 (clone GK1.5), CD3 (clone 17A2), I-A/I-E (clone M5/114.15.2), CD40 (clone 3/23), CD80 (clone 16-10A1), CD11c (N418), CD11b (clone M1/70), Foxp3 (clone MF-14 and REA788), and IFN-γ (clone XMG1.2) were used. For intracellular FOXP3 and cytokines, staining was done following the manufacturer protocol using Foxp3 Fix/Perm buffer kit (Biolegend) or FoxP3 Staining Buffer Set (miltenyi Biotec). Mouse Fc block (anti CD16/CD32 clone 93) was used prior to staining with markers antibodies. Antibodies were purchased from BioLegend and Miltenyi Biotec. Viobility 405/452 Fixable Dye (Miltenyi Biotec) was used for the discrimination of dead cells. Data were acquired on Fortessa (BD) or MACSQuant Analyzer 10 (Miltenyi Biotec) flow cytometers and analyzed using FlowJo software (Ashland).

### Statistical analysis

Data were analyzed with Graphpad Prism software (Graphpad), and *P* values of < 0.05 were considered to be statistically significant and indicated as *, *P* < 0.05; **, *P* < 0.01; and ***, *P* < 0.001.

## Results

### TTFields treatment potentiates immunogenic cell death (ICD) in cancer cells

**Fig. 1:**
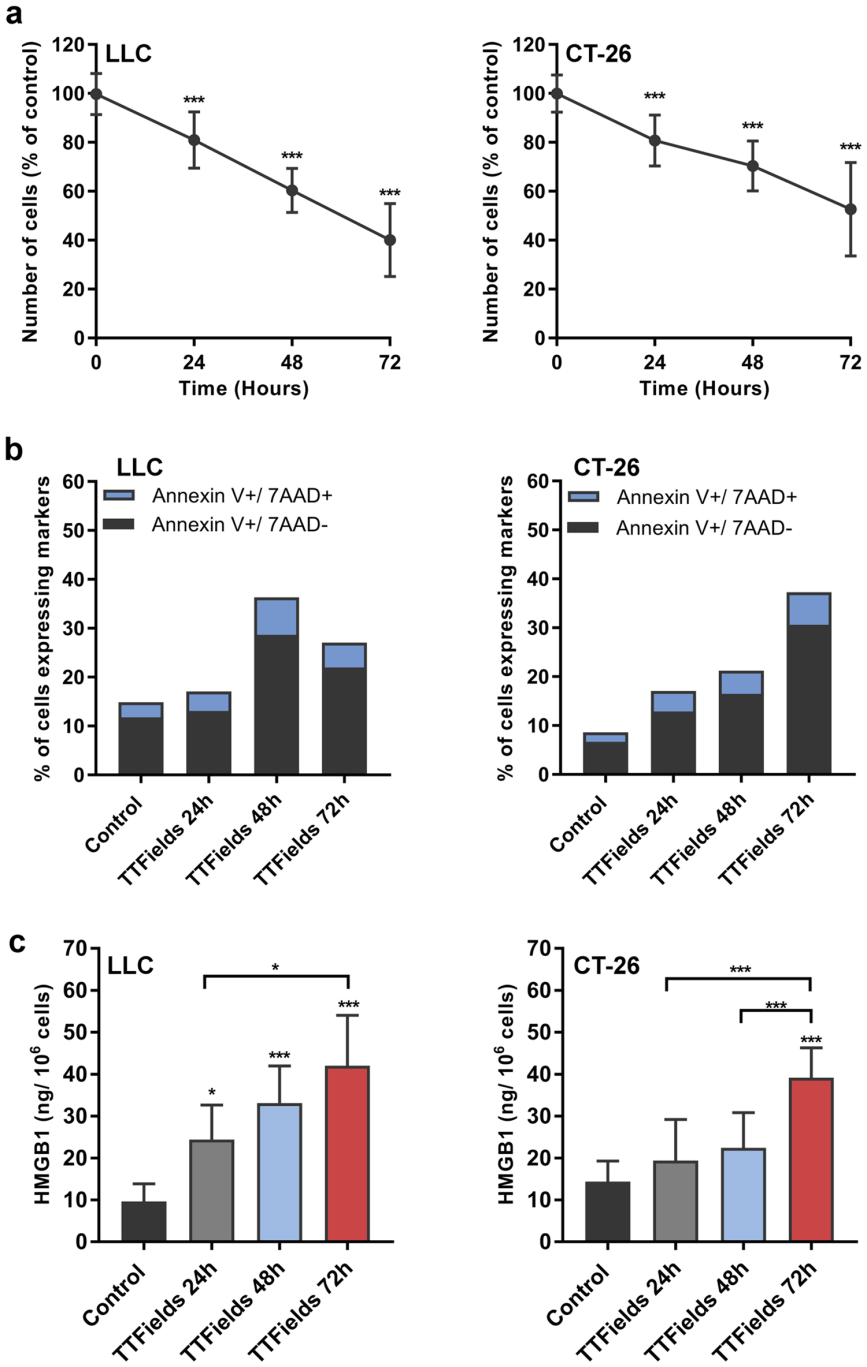
TTFields kill cancer cells by triggering apoptosis followed by extracellular release of HMGB1. LLC-1 and murine CT-26 colon carcinoma cells were treated with TTFields for 24–72 h at optimal frequency. **a** Effect of TTFields treatment on cell counts. *N* ≥ 3, and data are presented as mean ± SD. **b** Elevation of percentage of early apoptotic cells (Annexin V +/7AAD-) and late apoptotic cells (Annexin V+/7AAD+) following treatment with TTFields. *N* ≥ 3. **c** Release of HMGB1 was monitored using ELISA assay. *N* ≥ 3, and data are presented as mean ± SD. *P* values were determined using one-way ANOVA followed by Dunnett’s post-test. *P < 0.05; **P < 0.01; ***P < 0.001

To determine whether TTFields application promote ICD, murine Lewis lung carcinoma (LLC-1), murine colon carcinoma (CT-26), murine ovarian surface epithelial (MOSE-L), human hepatocellular carcinoma (HEPG2), and human lung squamous cell carcinoma (H520) cells were treated with TTFields for 24–72 h, each at its optimal frequency [9]. The tested cell lines demonstrated reduction in cell counts (Fig. 1a, Supplementary Fig. 1a, b) as well as induction of apoptosis as indicated by staining with annexin V and the vital dye, 7-Aminoactinomycin D (7AAD; Fig. 1b, Supplementary Fig. 1c–e). Since apoptosis is considered to be immunologically quiescent [10, 11], we then examined whether TTFields-induced cell death is accompanied by the release of the alarmin high-mobility group box 1 (HMGB1) protein. Indeed, upon treatment with TTFields the tested cell lines exhibited significant secretion of HMGB1, which, in accordance with cell death, was also dependent on treatment duration (Fig. 1c, Supplementary Fig. 1f–g).

**Fig. 2:**
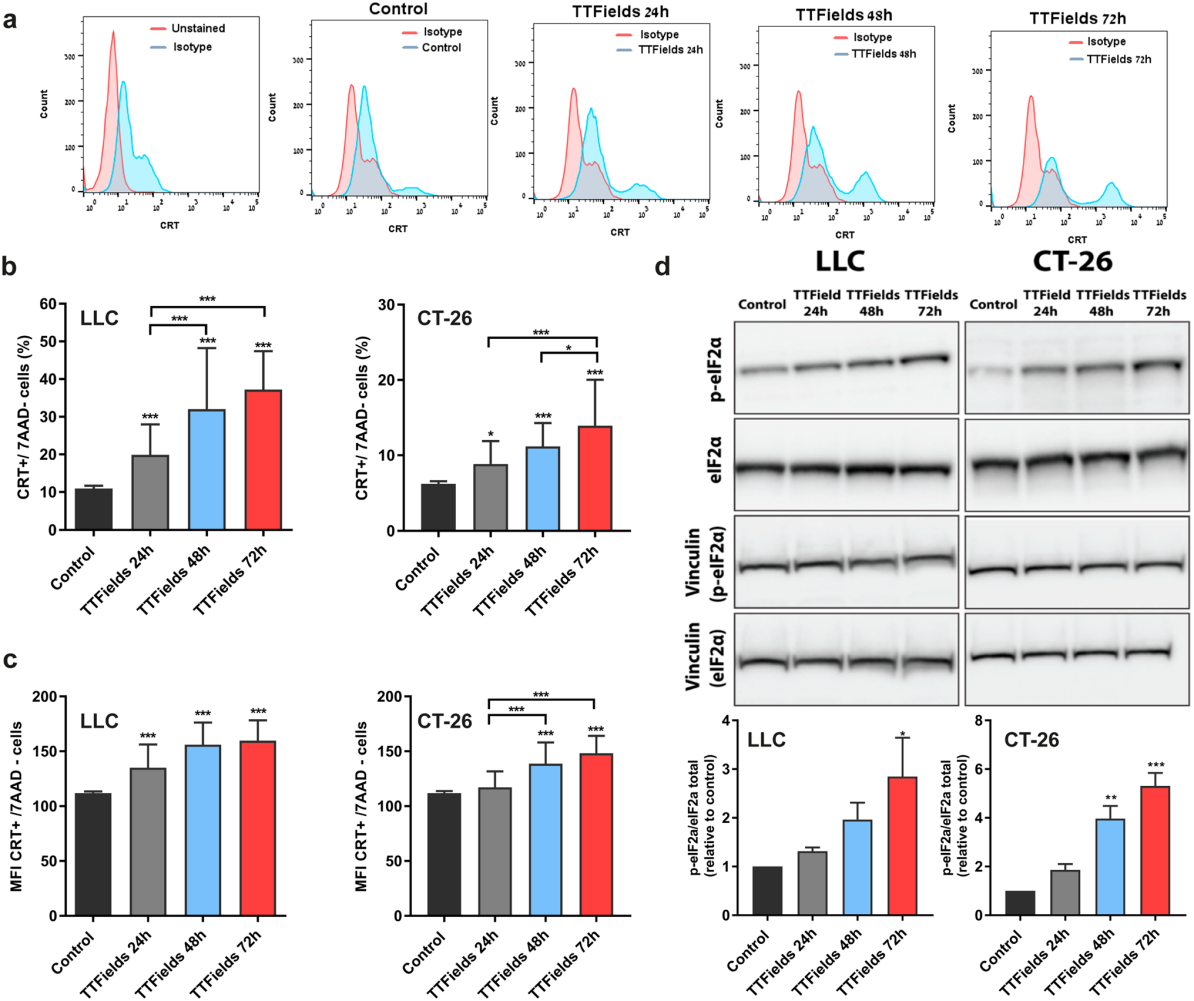
TTFields application mediate cell surface exposure of calreticulin. LLC-1 and CT-26 cells were treated with TTFields for 24–72 h at optimal frequency. A quantitative analysis of CRT surface exposure was performed using flow cytometry. **a** Representative plots of flow cytometry analysis of CRT, **b** CRT surface exposure among viable cells (7AAD-). **c** Median fluorescence intensity of CRT in viable cells. In **b** and **c***N* ≥ 3, and data are presented as mean ± SD, **d** Upper panel—Immunoblot analysis of peIF2α. Lower panel—densitometric analysis (arbitrary units normalized on the expression of the housekeeping protein Vinculin). *N* ≥ 3, and data are presented as mean ± SEM; *P* values were determined using one-way ANOVA followed by Dunnett’s post-test. *P < 0.05; **P < 0.01; ***P < 0.001

We next analyzed the effects of TTFields on another prominent prerequisite of ICD, i.e., translocation of the chaperone CRT to the cell surface of dying cells [12]. A quantitative analysis of CRT surface exposure revealed that TTFields treatment induced enhanced surface exposure of CRT in all tested cell lines. Specifically, we saw a significant increase in both percentages of viable CRT positive cells (Fig. 2a, b, Supplementary Fig. 2a, b) and cell surface distribution of CRT, as evaluated by the differences in median fluorescence intensity (MFI; Fig. 2c, Supplementary Fig. 2c, d). As seen with the induction of apoptosis and the release of HMGB1, CRT surface exposure was also dependent on treatment duration. To better understand why TTFields-induced cell death activates the exposure of CRT, and in accord with the mandatory role of ER stress for this process, we examined whether TTFields treatment leads to ER stress response [13, 14]. Our results demonstrate that under TTFields application, the translation initiation factor eIF2α becomes phosphorylated (Fig. 2d). These results demonstrate that TTFields induce ER stress response, which may be the trigger to the observed CRT translocation to the cell surface [15].

### Upregulation of autophagy in response to TTFields results in secretion of ATP

**Fig. 3:**
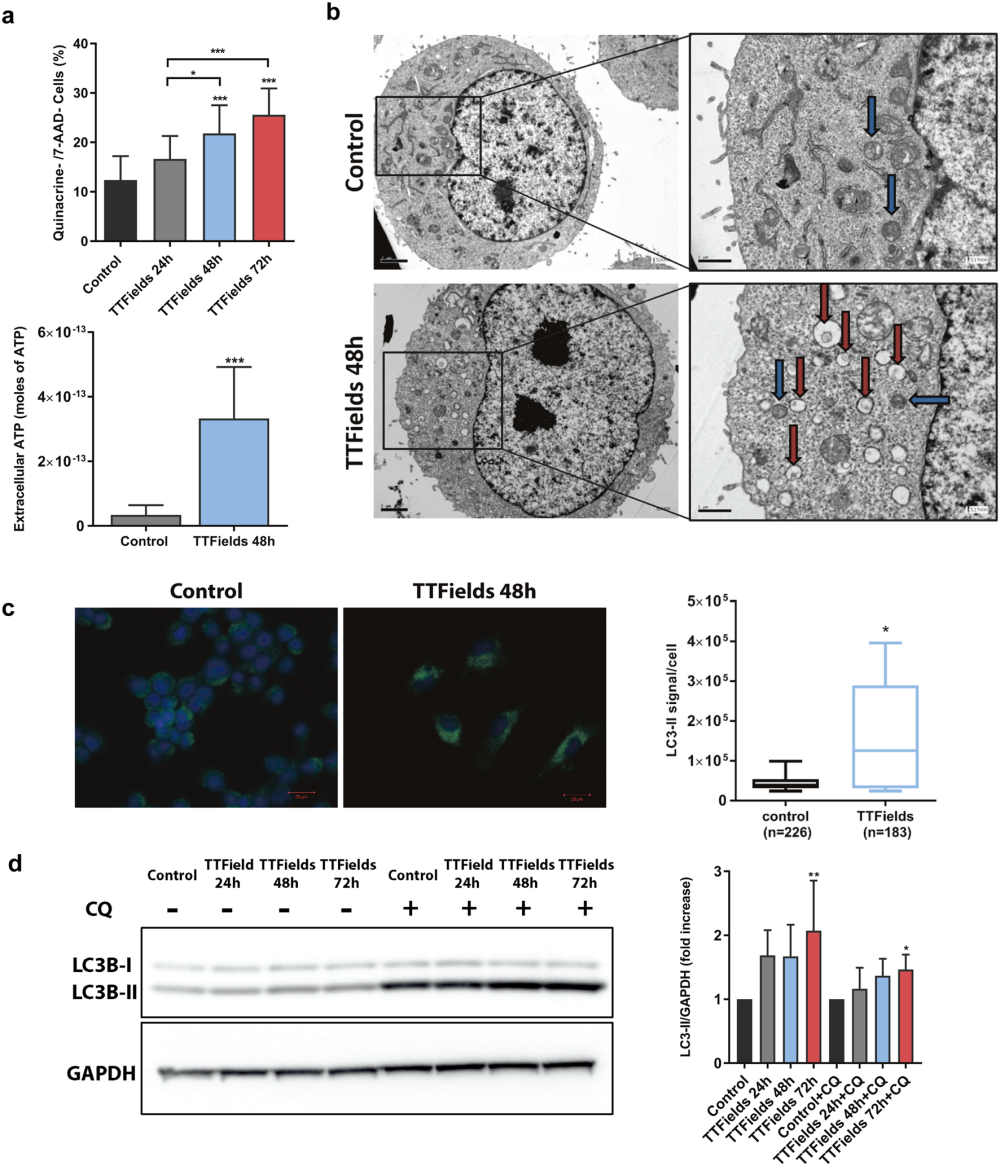
TTFields induce autophagy-dependent reduction in intracellular ATP levels. LLC-1 cells were treated with TTFields for 24–72 h at optimal frequency. **a** Flow cytometry measurements of quinacrine staining in viable cells (7AAD-) as indicator of intracellular ATP levels (Upper panel). *N* ≥ 3, and data are presented as mean ± SD. Alternatively, ATP release induced by TTFields was measured by enzymatic methods (lower panel). *N* = 2, the results are from one representative experiment, and data are presented as mean ± SD. **b** Ultrastructural scanning transmission electron microscope analysis of LLC-1 cells treated with TTFields for 48 h. Autophagosomes (Red arrows) and autolysosomes (Blue arrows) are indicated. Magnification ×6000, ×15000. **c** Immunofluorescence staining for LC3 (green) and DAPI (blue; left panel). Magnification ×40. Quantification of LC3II signal per cell (right panel). *N* = 4, and the results are reported as mean ± SD of pooled independent experiments. **d** LLC-1 cells were either left untreated, or treated with TTFields for 24–72 h. CQ (20 µM) was added 4 h before cells were collected. Samples were immunoblotted for LC3 and GAPDH. *N* = 2, and the results are reported as mean ± SD of pooled independent experiments. *P* values were determined using one-way ANOVA followed by Dunnett’s post-test for (**a**-upper panel, **d**) or unpaired two-tailed t test for (**a**-lower panel, **c**). *P < 0.05; **P < 0.01; ***P < 0.001

Along with cell surface exposure of CRT and the release of HMGB1, the secretion of adenosine triphosphate (ATP) by dying cells constitutes one of the hallmarks of ICD [16]. To further explore the observation that TTFields application potentially induces ICD in replicating cells, we examined ATP secretion by treated cells. We performed flow cytometry measurements of quinacrine staining in viable cells as an indicator of intracellular ATP levels in cells exposed to TTFields [17]. Cells treated with TTFields exhibited reduced quinacrine staining (Fig. 3a upper panel, Supplementary Fig. 3). This reduction was also accompanied by secretion of ATP (Fig. 3a lower panel). Since autophagy is required for the optimal release of ATP from dying cells, we investigated whether TTFields affect autophagy in the treated cells [18, 19]. Transmission electron microscopy analysis revealed the accumulation of vacuoles with the typical morphological appearance of autophagic structures containing cytosolic materials (Fig. 3b; red arrows) in treated cells. To further confirm these findings, lipidated LC3 (LC3-II) levels were used to monitor autophagy using immunoblotting and immunofluorescence microscopy. Increased punctate distribution of LC3-II was observed following TTFields treatment (Fig. 3c). The observed LC3-II accumulation suggests either an increase in the number of autophagic events or a block in autophagosome degradation. To assess this, we performed immunoblot analysis of LC3-II in the presence or absence of the lysomotropic agent chloroquine (CQ) [20]. The addition of CQ led to increased LC3-II levels in TTFields-treated cells, demonstrating enhanced autophagic flux, which can explain the induction of ATP release (Fig. 3d).

### Cancer cells that die under TTFields application induce DC maturation and leukocyte recruitment

**Fig. 4:**
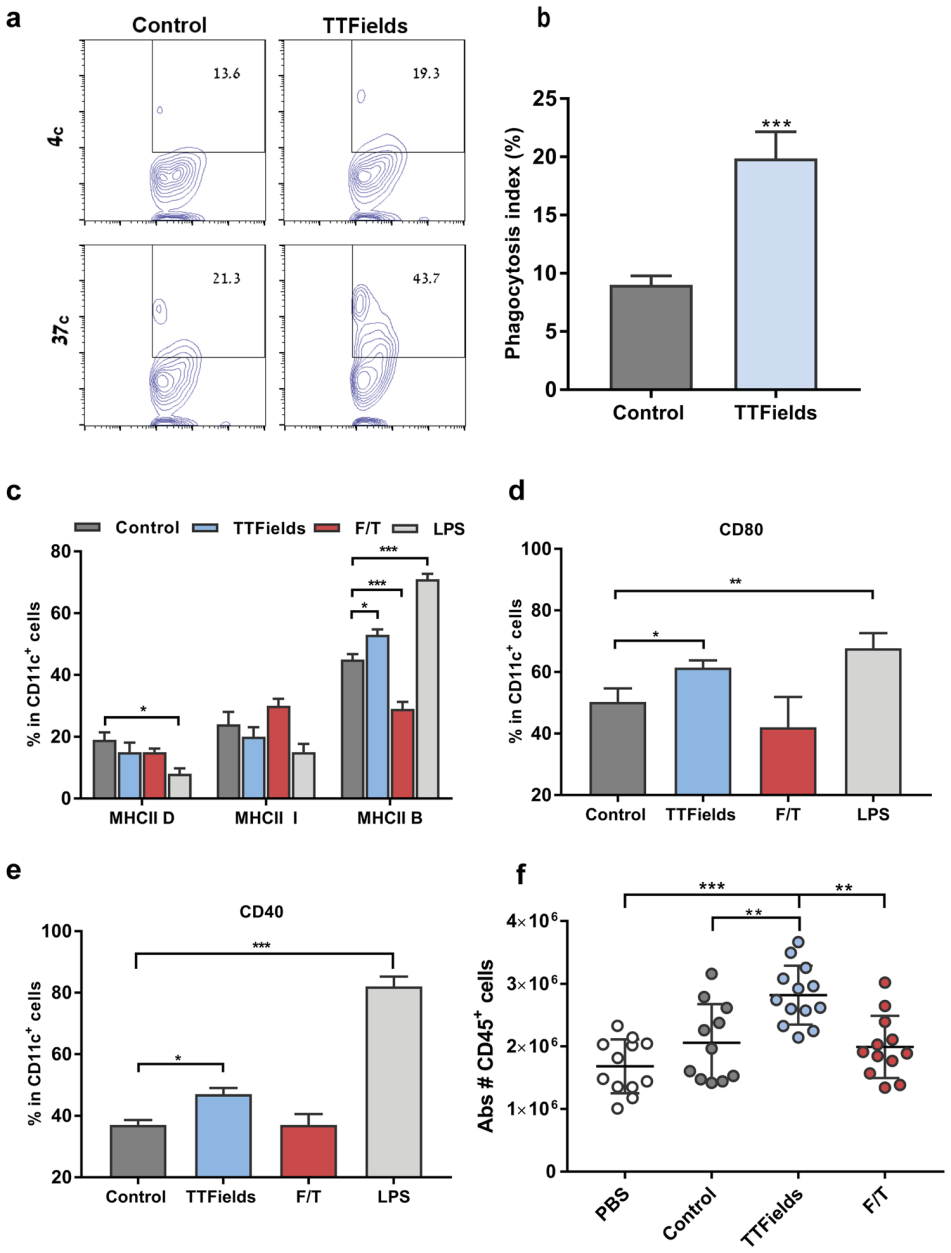
Culture of TTFields-treated cancer cell suspension induces maturation of bone marrow-derived dendritic cells. **a**, **b** Co-culture of cell tracker deep red labeled, untreated, or TTFields-treated LLC-1 cells and BMDCs. CD11c+Deep Red+events were quantified using flow cytometry. **a** Representative flow cytometry plot and **b** quantification of phagocytosis index. *N* = 3, and data are presented as mean ± SD. **c–e** Co-culture of untreated, TTFields-treated, F/T-treated, or LPS-treated LLC-1 cells and BMDCs. BMDCs maturation phenotype was assessed using flow cytometry. Quantification of activation markers: **c** MHC II (D-dim, I-intermediate, and B-bright expression), **d** CD80, and **e** CD40. *N* ≥ 4, and data are presented as mean ± SEM. **f** LLC-1 cells were either left untreated or treated with TTFields- (48 h), or F/T-treated cells and were intraperitoneally injected into wild-type C57/Bl6 mice. Equal volumes of PBS were injected in mice as negative controls. Peritoneal exudate cells were collected 48 h later and analyzed using flow cytometry. Graphs represent the number of CD45 + cells in peritoneal exudate cells. *N* = 2, bars indicate mean of two pooled independent experiments with 5–7 mice per group. Each circle represents one mouse. *P* values were determined using unpaired two-tailed t test for **(b)** or one-way ANOVA followed by Dunnett’s post-test **(c–f)**. *P < 0.05; **P < 0.01; ***P < 0.001

We next evaluated the actual potential of cells that die during TTFields application to promote phagocytosis by DCs by challenging bone marrow-derived DCs (BMDCs) with LLC-1 cells that were treated with TTFields for 48 h. TTFields-treated cells were effectively phagocytosed by BMDCs, while untreated control cells did not show similar outcome (Fig. 4a, b). To assess the functional status of these BMDCs, we also analyzed the surface expression of the activation markers MHC class II (MHC II), CD40, and CD80. Lipopolysaccharide (LPS)-treated cells served as a positive control, and cells subjected to freeze–thaw cycles (F/T cells) served as negative control. After overnight culture with control, TTFields, or F/T-treated LLC-1 cell suspension, these markers were found to be upregulated in BMDCs that were co-cultured with TTFields-treated cells. This upregulation was observed for all the costimulatory molecules analyzed (Fig. 4c–e, Supplementary Fig. 4). Finally, to test the ability of TTFields-treated cells to attract immune cells in vivo, we evaluated leukocytes recruitment in mice following intraperitoneal injection of: live, F/T-treated, or TTFields-treated cells. Intraperitoneal injection of TTFields-treated cells induced significant recruitment of leukocytes (CD45+), as compared to control or F/T-treated cells (Fig. 4f). Together, these results indicate that TTFields-treated cells promote phagocytosis by DCs, DC maturation in vitro, and leukocyte recruitment in vivo.

### Combining TTFields with anti-PD-1 enhances antitumor immunity and results in increased tumor control in vivo

**Fig. 5:**
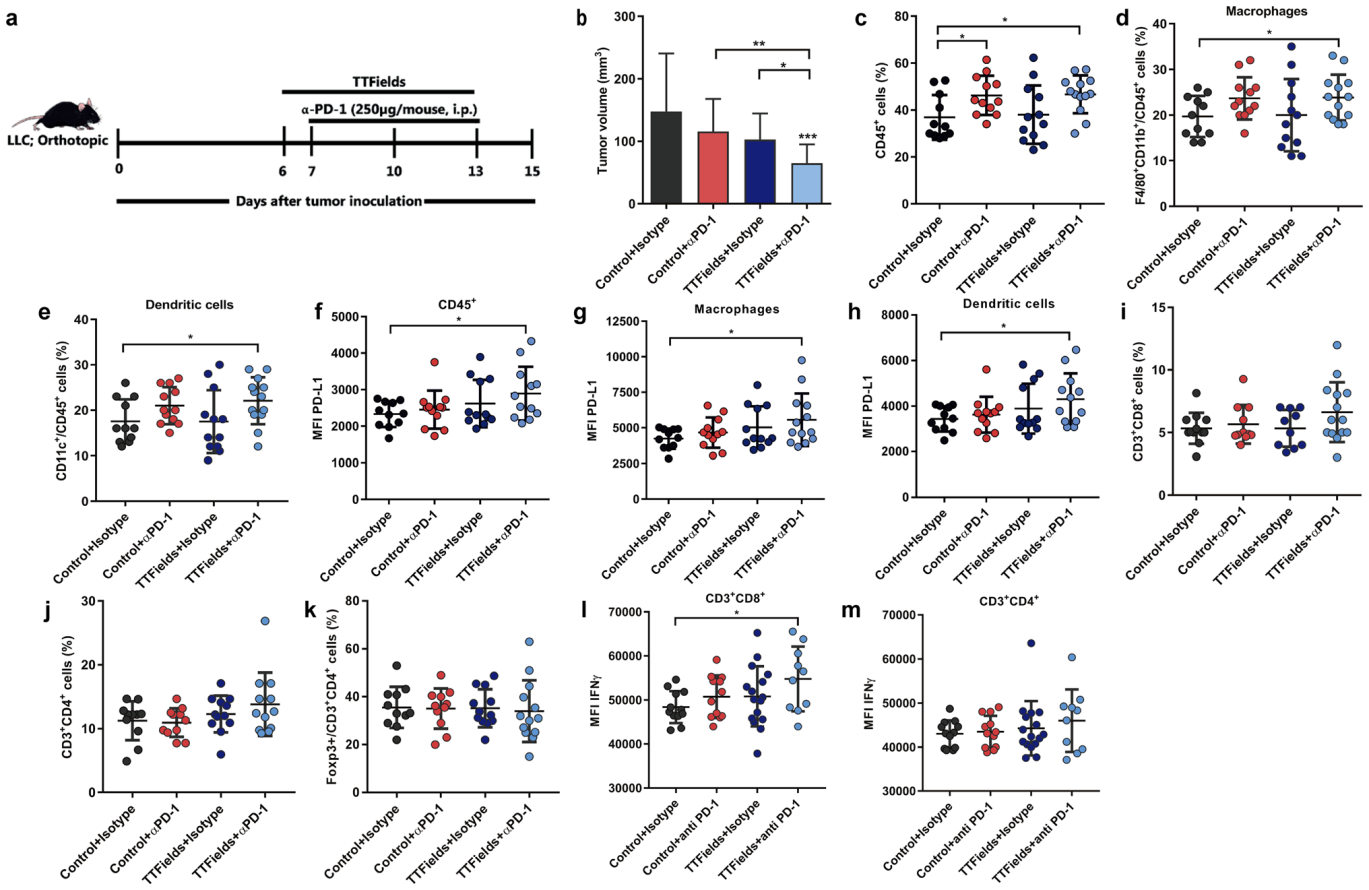
TTFields in combination with anti-PD-1 are therapeutically effective in murine lung cancer model. Ten-12-week-old male C57Bl/6 mice were injected directly into the lungs with Lewis lung carcinoma (LLC-1; 3 × 10^3^ cells). **a** Application of TTFields to the mouse lungs was initiated 6 days afterward and was maintained for 7 days. Mice received an I.P. injection of anti-PD-1 (αPD-1) or Rat IgG2a, as indicated in the scheme. **b** At the end of the experiment, tumor volume was measured using Vernier calipers. *N* = 3, and the results are reported as mean ± SD of three pooled independent experiments with total of 5–8 mice per group. Tumors were harvested and analyzed for the percentages (**c**–**e**) and PD-L1 expression levels (**f**–**h**) of tumor (**c**, **f**) CD45+ cells, **d**, **g** dendritic cells (CD45+CD11c+), **e**, **h** macrophages (CD45+CD11b+F4/80+) using flow cytometry. Percentages of **i** CD3+CD8+, **j** CD3+CD4+, and **k** Foxp3+CD3+CD4+TILs. **l**, **m** TILs were harvested from LLC-1 tumor-bearing mice and stimulated with anti-CD3 and anti-CD28 before intracytoplasmic cytokine staining. Expression levels of IFN-γ+in CD3+CD8+ (**l**) and CD3+CD4+ (**m**). *N* = 2, and bars indicate mean of two pooled independent experiment with 5–8 mice per group. Each circle represents one mouse. *P* values were determined using the Kruskal–Wallis test followed by a Dunn’s post-test for (**b**) or unpaired two-tailed t test for (**c**–**m**). *P < 0.05; **P < 0.01; ***P < 0.001.* MFI* Median fluorescence intensity

To evaluate the effect of concurrent application of TTFields and anti-PD-1 therapy on normal lung tissue, non-tumor-bearing C57Bl/6 mice were treated with TTFields, anti-PD-1, or the combination of the two modalities. Histopathological analysis of the lungs determined that there were no pathological changes in the lungs from the different treatment groups and that the leukocytes level was also within the normal limits in all treatment groups (Supplementary Fig. 5). To further evaluate the effect of concurrent application of TTFields and anti-PD-1 therapy on tumors, C57Bl/6 mice orthotopically implanted with LLC-1 cells were treated with TTFields (Supplementary Fig. 6), anti-PD-1, or the combination of the two modalities (Fig. 5a). Mice treated with anti-PD-1 and TTFields monotherapies demonstrated decreased tumor volume as compared to the control group, although statistical significance was not reached (Fig. 5b). The combined treatment of TTFields and anti-PD-1 led to a significant decrease in tumor volume as compared to all the other groups. A significant increase in leukocyte infiltration (CD45+) was observed in both groups receiving anti-PD-1 injections (Fig. 5c). We next characterized the frequency of specific myeloid populations to the tumors. Specifically, we found a significantly higher frequency of macrophages (CD45+/CD11b+/F4/80+) and DCs (CD45+/CD11c+) in tumors from mice that were concomitantly treated with TTFields and anti-PD-1. There were no significant differences in the frequency of macrophages and DCs between mice treated with TTFields alone or anti-PD-1 alone and the control group. A trend toward increase in these cell populations was observed in mice treated with anti-PD-1 injections (Fig. 5d, e). We also examined whether PD-L1 expression levels, associated with response to anti-PD-1 therapy and adaptive immune resistance, had changed in these myeloid populations following the different treatments. The PD-L1 expression levels of tumor-infiltrating CD45+ cells were increased in tumors from mice treated with TTFields in combination with anti-PD-1 as compared to the control group, suggesting elevated inflammatory response in these tumors. No significant differences were observed between the other groups (Fig. 5f). Specifically, a significant upregulation of surface PD-L1 expression was demonstrated in macrophages and DCs in tumors from mice treated with anti-PD-1 and TTFields, suggesting an adaptive immune attempt to limit the inflammatory response elicited by the combined treatment (Fig. 5g, h) [21]. There were no significant differences in the PD-L1 levels of macrophages and dendritic cells between mice treated with TTFields or anti-PD-1 monotherapies and the control mice injected with the isotype antibody. Taken together, these results suggest that the combination of TTFields and anti-PD-1 augmented the immune response resulting in improved tumor control.

**Fig. 6:**
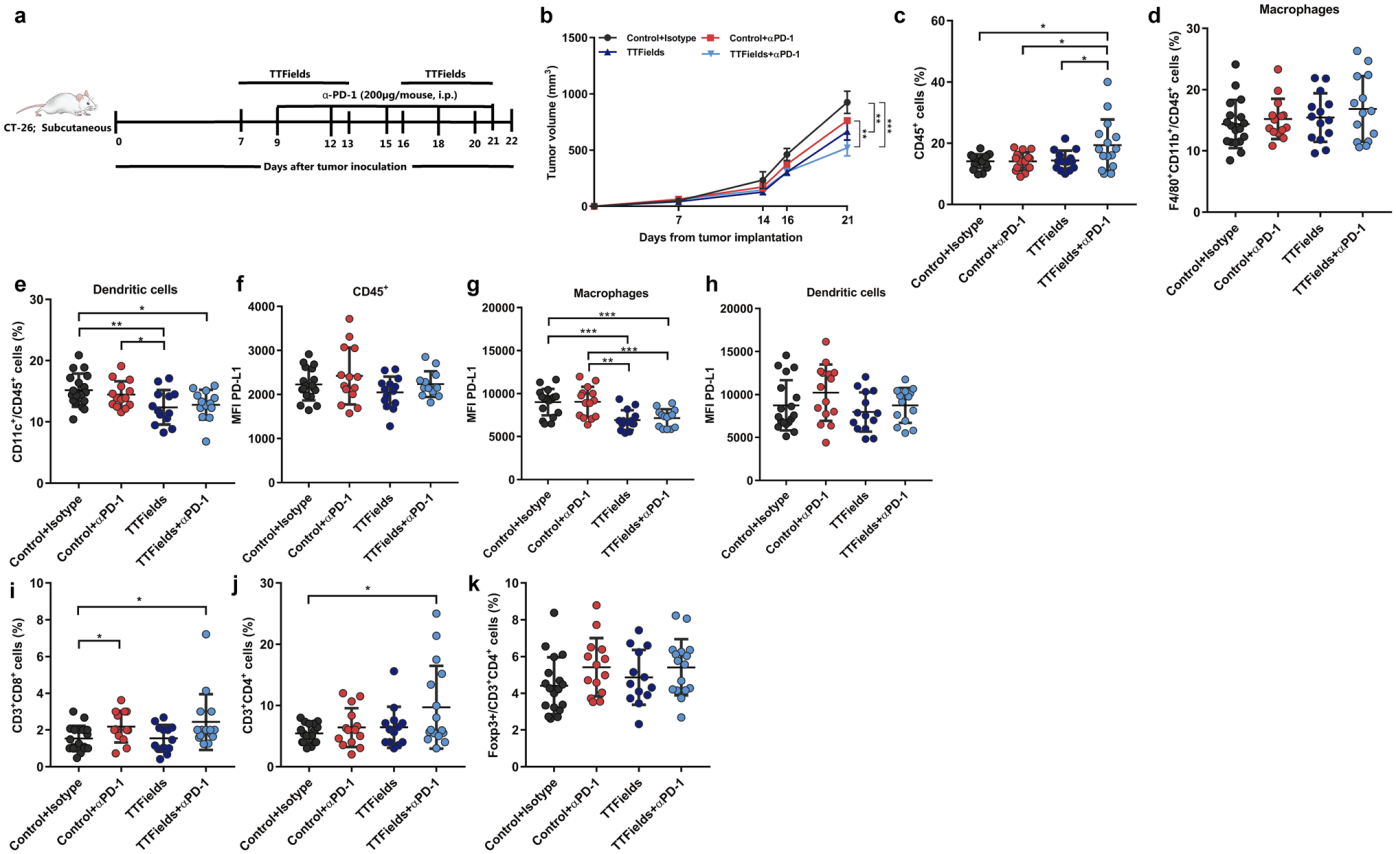
TTFields in combination with anti-PD-1 are therapeutically effective in murine colon cancer model. **a** Ten-week-old female Balb/c mice bearing 60 mm^3^ subcutaneous CT-26 tumors were treated with TTFields for 14 days, with a 3-day break (days 13–16). Mice received an I.P. injection of anti-PD-1 (αPD-1) or Rat IgG2a, as indicated in the scheme. **b** At the end of the experiment, tumor volume was measured using Vernier calipers. *N* = 2, and the results are reported as mean ± SD of two pooled independent experiments with total of 5–11 mice per group. Tumors were harvested and analyzed using flow cytometry for the percentages **(c–e)** and PD-L1 expression levels (**f**–**h**) of tumor **(c, f)** CD45+ cells, **d, g** dendritic cells, **e, h** macrophages. Percentages of **i** CD3+CD8+, **j** CD3+CD4+, and **k** Foxp3+CD3+CD4+TILs. *P* values were determined using two-way ANOVA with Tukey’s post-test for **(b)** or unpaired two-tailed t test for **(c–k)**. *P < 0.05; **P < 0.01; ***P < 0.001

To directly address whether treatment with TTFields plus anti-PD-1 promotes antitumor immunity, we first tested how the presence of TILs was affected by treatment with the different treatment modalities. We found that treatment with TTFields or anti-PD-1 alone had no effect on the frequency of CD8+ and CD4+TILs, while treatment with TTFields plus anti-PD-1 showed a trend toward an increased number of these cells, but this varied between experiments and did not reach statistical significance (Fig. 5i–k). We did not observe any significant changes in the levels of regulatory CD4+Foxp3+TILs between the different treatment groups. TILs functionality was tested in tumor-isolated lymphocytes following in vitro stimulation with anti-CD3 and anti-CD28 (Fig. 5l, m). We found that the combined treatment of TTFields and anti-PD-1 led to a significant increase in IFN-γ production in cytotoxic CD8+TILs (Fig. 5l). The comparable results were obtained when we used the CT-26 tumor model (Fig. 6a), where mice treated with combination of TTFields and anti-PD-1 demonstrated decreased tumor volume as compared to all the other treatment groups (Fig. 6b). In addition, a significant increase in leukocyte infiltration was observed in tumors from mice receiving the combined treatment, as compared to all the other groups (Fig. 6c). The relative fraction of macrophages from tumor leukocytes did not change between the different treatment groups (Fig. 6d), and the relative fraction of DCs from tumor leukocytes has significantly decreased in tumors from mice receiving TTFields alone or the combined treatment (Fig. 6e), although their percentage of total tumor cells remain unaltered between the different treatments (data not shown). This can indicate that other populations of immune cells might contribute to the overall increase in CD45+ cells as observed in CT-26 tumors following treatment with TTFields and anti-PD-1. Furthermore, the PD-L1 expression levels decreased in macrophages from tumors treated with TTFields in combination with anti-PD-1 and TTFields alone (Fig. 6g). No significant differences were observed in DCs PD-L1 levels between the different treatment groups (Fig. 6h). While we did not observe significant change in the infiltration pattern of TILs from LLC-1 tumor, we identified significant increase in both CD8 and CD4 T-cells in the combined treatment tumors, and CD8 in tumors treated with anti-PD-1 alone (Fig. 6i, j, Supplementary Fig. 7). Similarly, to the LLC-1 model, we did not observe any effect on the frequency of CD4+Foxp3+TILs in any of the treatment groups (Fig. 6k).

## Discussion

In this study, we provide the first evidence for the immunostimulatory effects of TTFields-induced cell death. In a previous animal study, we had shown that TTFields treatment of orthotopically implanted VX2 renal tumors in rabbits led to a marked decrease in lung metastasis despite the fact that the field intensity in the lungs was too low to have an inhibitory effect on metastatic lesions [22]. Furthermore, a significant infiltration of immune cells to lung metastases of the TTFields-treated animals was observed. This outcome resembles the abscopal effect observed following radiotherapy and photodynamic therapy, suggesting an induction of immune-mediated antitumor effects [23, 24]. Additional evidence for the involvement of the immune system is clinical data, showing that patients concurrently treated with TTFields and high doses of the potent immunosuppressive drug dexamethasone exhibit poor outcome when compared to patients who used lower doses of dexamethasone [25, 26]. Our data extend these previous observations on the systemic effects of regional application of TTFields.

TTFields were shown to lead to mitotic catastrophe resulting in the formation of abnormal aneuploid progeny [6]. Hyperploidy was suggested as a driver of ER stress response and subsequent CRT exposure [27]. We show that TTFields application resulted in phosphorylation of eIF2α, a cardinal hallmark of the ER stress response, and the exposure of CRT at the cell surface [15]. These results demonstrate that TTFields, similar to a growing list of anticancer treatment modalities, contribute to cancer immunosurveillance by hyperploidy-induced ER stress and CRT exposure [12, 27, 28]. Aneuploidy can also stimulate autophagy, which is required for the immunogenicity of dying cells [29]. Autophagy was elevated following TTFields application, leading to ATP efflux in treated cells. In this regard, ATP secretion, which serves as a “find me” signal for apoptotic cells, could provide a possible explanation for the enhanced recruitment of lymphocytes into the peritoneal cavity of mice injected with TTFields-treated cells [30].

HMGB1 release from dying tumor cells, which is required for the processing and cross-presentation of antigens, serves as another hallmark of ICD [31, 32]. We show that cancer cells release HMGB1 upon treatment with TTFields. Furthermore, our experiments report the first demonstration that cells that die during TTFields application can promote DCs maturation, thereby providing evidence for the functionality of the released HMGB1 [33]. While TTFields were demonstrated to directly interfere with the mitotic events of cancer cells, the enhanced immune response may demonstrate TTFields’ role in treating distant metastasis outside TTFields effective range, through an abscopal effect.

Our in vivo data demonstrate that combining TTFields with anti-PD-1 may enhance antitumor immunity and result in increased tumor control as compared to either therapy alone. TTFields-treated cells successfully recruited leukocytes into the peritoneal cavity. However, this recruitment was not observed at the tumor level when TTFields were applied as a monotherapy. This suggests that while TTFields may be efficient at releasing damage-associated molecular patterns (DAMPs), the immunosuppressive tumor microenvironment may hamper the development of therapeutically effective antitumor immune responses. Treatment with anti-PD-1 monotherapy resulted in limited reduction in tumor volume in both models, stressing the added benefit of TTFields as a stimulant of immunogenic response.

The combined treatment of TTFields with anti-PD-1 results in several changes in the tumor microenvironment. First, increased infiltration of leukocytes and an obvious existence of T-cell-mediated antitumor effect were observed, albeit not accompanied with a significant increase in TILs infiltration in the LLC-1 model. This finding may be explained by the short treatment duration in this model relative to the CT-26 model. Second, the elevation in PD-L1 density in leukocytes from LLC-1 tumors in the combined treatment group may have resulted from increase in IFN-γ production in CD8+ cells in the tumor milieu [34, 35]. Similarly, increases in IFN-γ levels in these tumors could also be a potential explanation for the elevation in the percentage of dendritic cells and macrophages populating the tumors treated with the combination therapy [36]. In contrast, the combined treatment of TTFields and anti-PD-1 did not result in changes in PD-L1 density in leukocytes from CT-26 tumors, and PD-L1 density was reduced in macrophages from tumors treated with TTFields monotherapy or the combination of the two modalities. This finding can be attributed to the long-term treatment duration in this model and is in line with a recent study by Capasso et al., showing that PD-L1 expression is increased in early stages of treatment with anti-PD-1 and is decreased in later stages [37]. It should also be noted that CT-26 cancer cells express high levels of PD-L1 on their surface while LLC-1 cancer cells do not express PD-L1; therefore, changes in PD-L1 expression in the tumor microenvironment can also potentially be affected by the PD-L1 expression by the cancer cells as well as by drug treatment schedule.

Another point that should be considered is the diversity of the preexisting immune cell subpopulations between the two tumor models [38, 39]. The fact that the final tumor volumes in the combined treatment group are about tenfold higher in the CT-26 model compared to the LLC-1 model could by itself influence the immune cell subpopulations, as it was shown in CT26 tumors that cytotoxic T-cells increase in density as tumor volume increases [38]. While the current study focused on specific subpopulations, it is possible that additional immune infiltrates could play a significant role in dictating the response to the combination therapy. For instance, high prevalence of immunosuppressive populations (e.g., myeloid-derived suppressor cells, tumor-associated macrophages) has been shown to impair responses to immunotherapies [40–42]. Future studies focusing on high-dimensional phenotypic analysis of the complexity within the tumor milieu are likely to reveal the effects of TTFields on the functionality of additional immune cell entities, as well as provide new framework for design of combination therapies in different tumor models.

Taken together, we provide evidence on the immune-stimulatory effects of TTFields-induced cell death. Our data also show a therapeutic advantage for combining TTFields and anti-PD-1, highlighting that this combination is a viable treatment regimen to enhance clinical outcome. The combination of TTFields with immune check point inhibitors is currently also being tested in a phase-3 clinical study (the LUNAR Trial—NCT02973789).

## Electronic supplementary material

Below is the link to the electronic supplementary material.

Supplementary material 1 (PDF 1461 kb)

## Abbreviations

ATP: Adenosine triphosphate
BMDCs: Bone marrow-derived DCs
CQ: Chloroquine
CRT: Calreticulin
DCs: Dendritic cells
F/T: Freeze–thawed
HMGB1: High-mobility group B1
ICD: Immunogenic cell death
LC3: Microtubule-associated protein 1A/1B-light chain 3
LPS: Lipopolysaccharide
LLC-1: Lewis lung carcinoma (LLC-1)
MFI: Median fluorescence intensity
MOSE-L: Murine ovarian surface epithelial
PDT: Photodynamic therapy
TILs: Tumor-infiltrating leukocytes
TTFields: Tumor-treating fields
7-AAD: 7-Aminoactinomycin D
