# Supplementary material for: Tumor-treating fields (TTFields) induce immunogenic cell death resulting in enhanced antitumor efficacy when combined with anti-PD-1 therapy
Source: Cancer Immunol Immunother. 2020 Mar 6;69(7):1191–204. doi: 10.1007/s00262-020-02534-7 (PMC7303058; doi:10.1007/s00262-020-02534-7)
Supplement: Supplementary file 1 — Supplementary material 1 (PDF 1461 kb) [file 262_2020_2534_MOESM1_ESM.pdf]

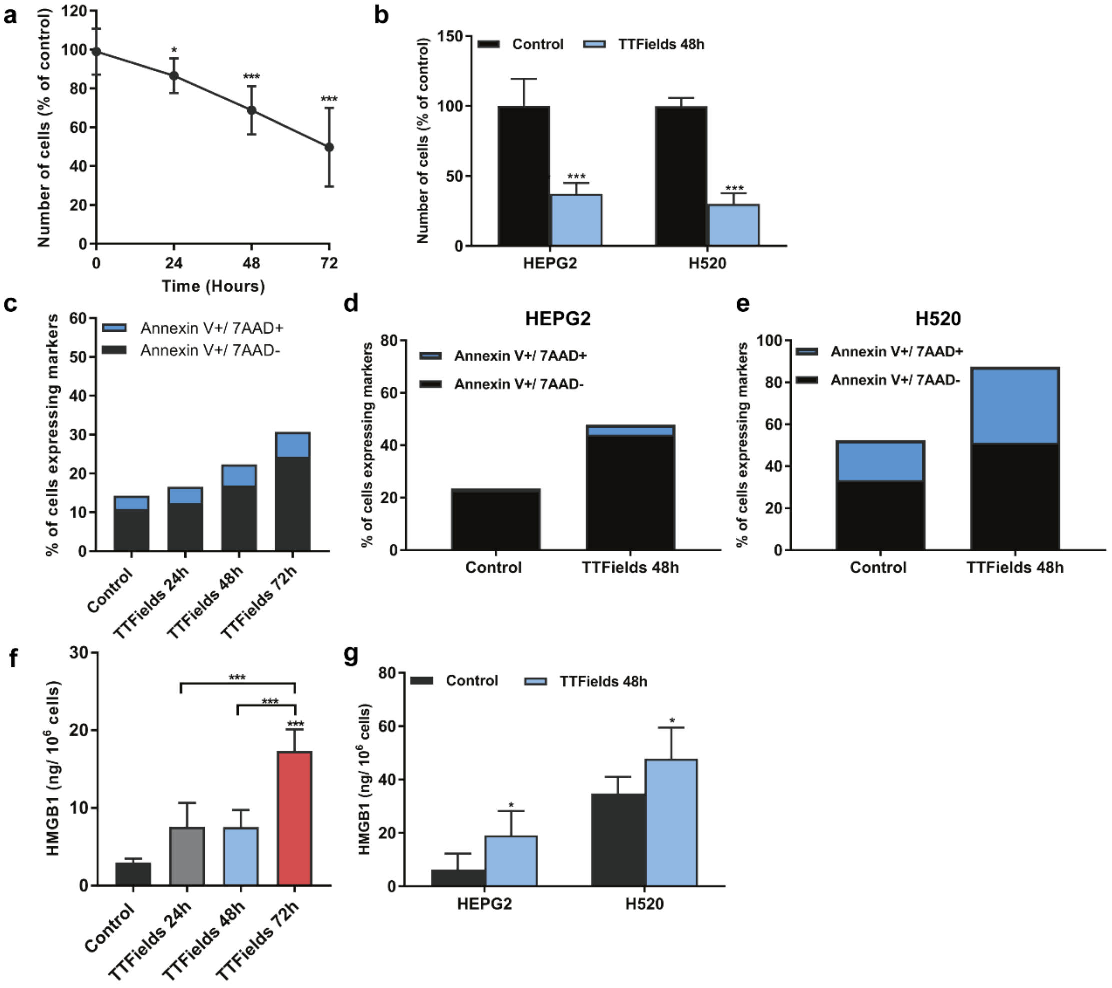


**Supplementary Figure 1. TTFields kill cancer cells by triggering apoptosis followed by extracellular release of HMGB1. (a)** MOSE-L cells were treated with TTFields for 24-72 hours at optimal frequency. **(b)** HEPG2 cells and H520 cells were treated with TTFields for 48 hours at optimal frequency. **(a-b)** Effect of TTFields treatment on cell counts are shown N≥3, and data are presented as mean ± SD. **(c-e)** Elevation of percentage of early apoptotic cells (Annexin V+ /7AAD-) and late apoptotic cells (Annexin V+ /7AAD+) following treatment with TTFields as indicated in **(a-b)** in MOSE-L cells **(c)**, HEPG2 cells **(d)**, and H520 cells **(e)**. N≥3. **(f)** MOSE-L cells, **(g)** HEPG2 and H520 cells were treated with TTFields as indicated in **(a-b)**. Release of HMGB1 following treatment was monitored using ELISA assay. N≥2 and data are presented as mean ± SD. p values were determined using 1-way ANOVA followed by Dunnett's post-test. *P < 0.05; **P < 0.01; ***P < 0.001

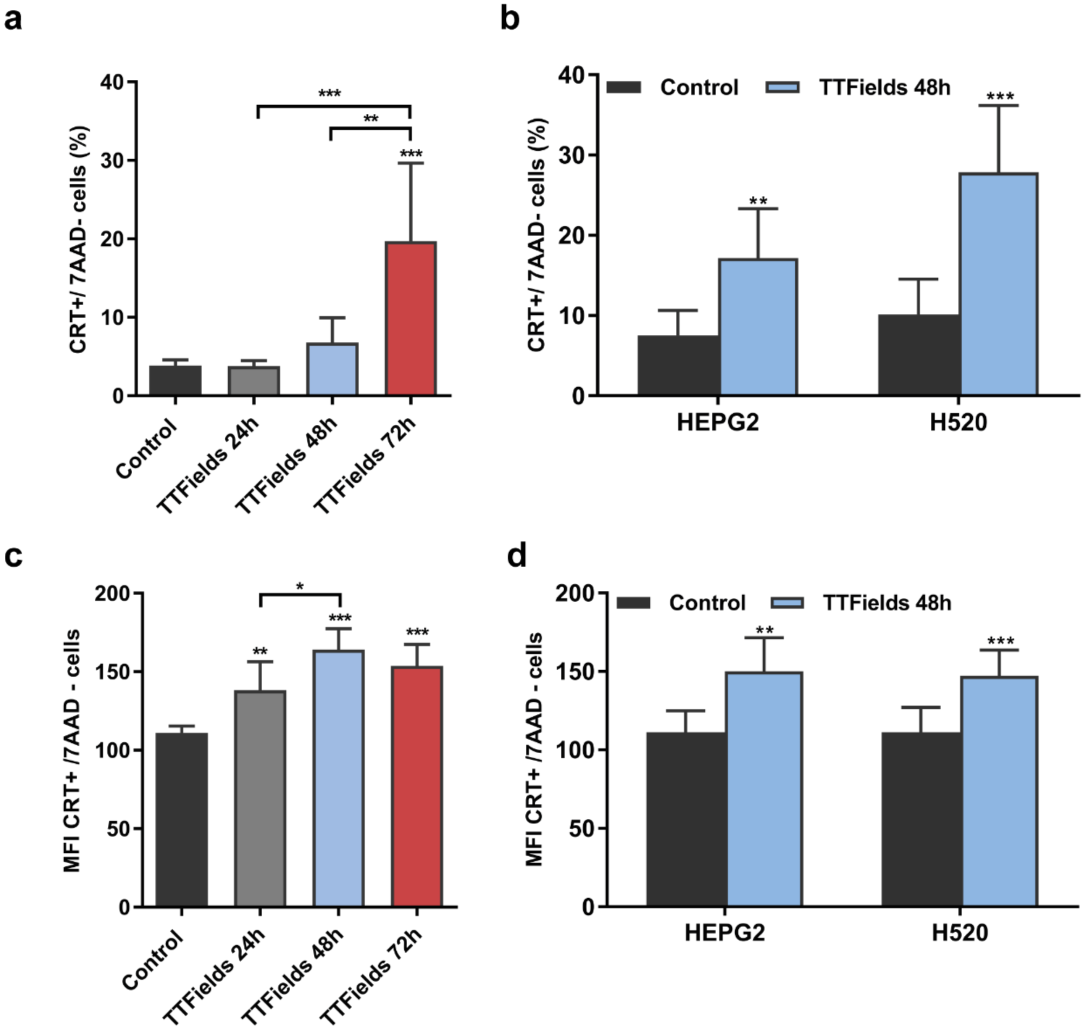


**Supplementary Figure 2. TTFields application mediate cell surface exposure of calreticulin. (a)** MOSE-L cells were treated with TTFields for 24-72 hours at optimal frequency. **(b)** HEPG2 cells and H520 cells were treated with TTFields for 48 hours at optimal frequency. **(a-d)** A quantitative analysis of CRT surface exposure was performed using flow cytometry. **(a-b)** CRT surface exposure among viable cells (7AAD-). **(c-d)** Median fluorescence intensity of CRT in viable cells. N≥2 and data are presented as mean ± SD. p values were determined using 1-way ANOVA followed by Dunnett's post-test. *P < 0.05; **P < 0.01; ***P < 0.001

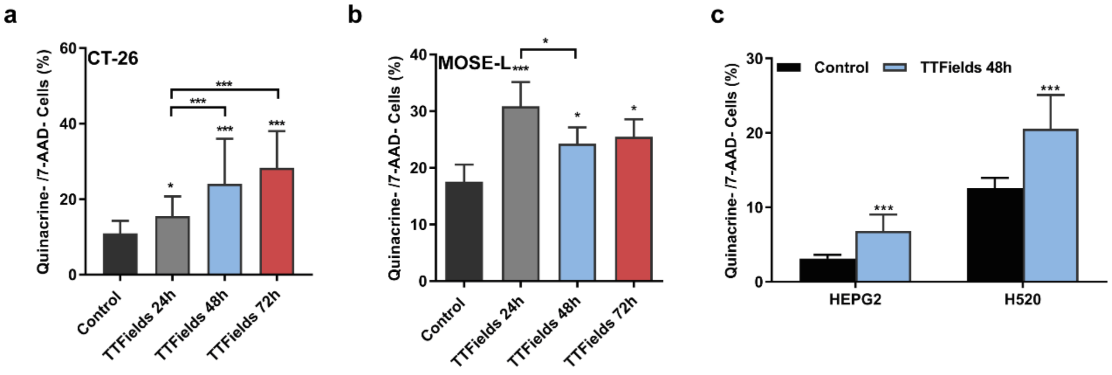


**Supplementary Figure 3**. **TTFields induces reduction in intracellular ATP levels. (a)** CT-26 and **(b)** MOSE-L cells were treated with TTFields for 24-72 hours at optimal frequency. **(c)** HEPG2 cells and H520 cells were treated with TTFields for 48 hours at optimal frequency. **(a-c)** Flow cytometry measurements of quinacrine staining in viable cells (7AAD-) as indicator of intracellular ATP levels. N≥2 and data are presented as mean ± SD. p values were determined using 1-way ANOVA followed by Dunnett's post-test. *P < 0.05; **P < 0.01; ***P < 0.001

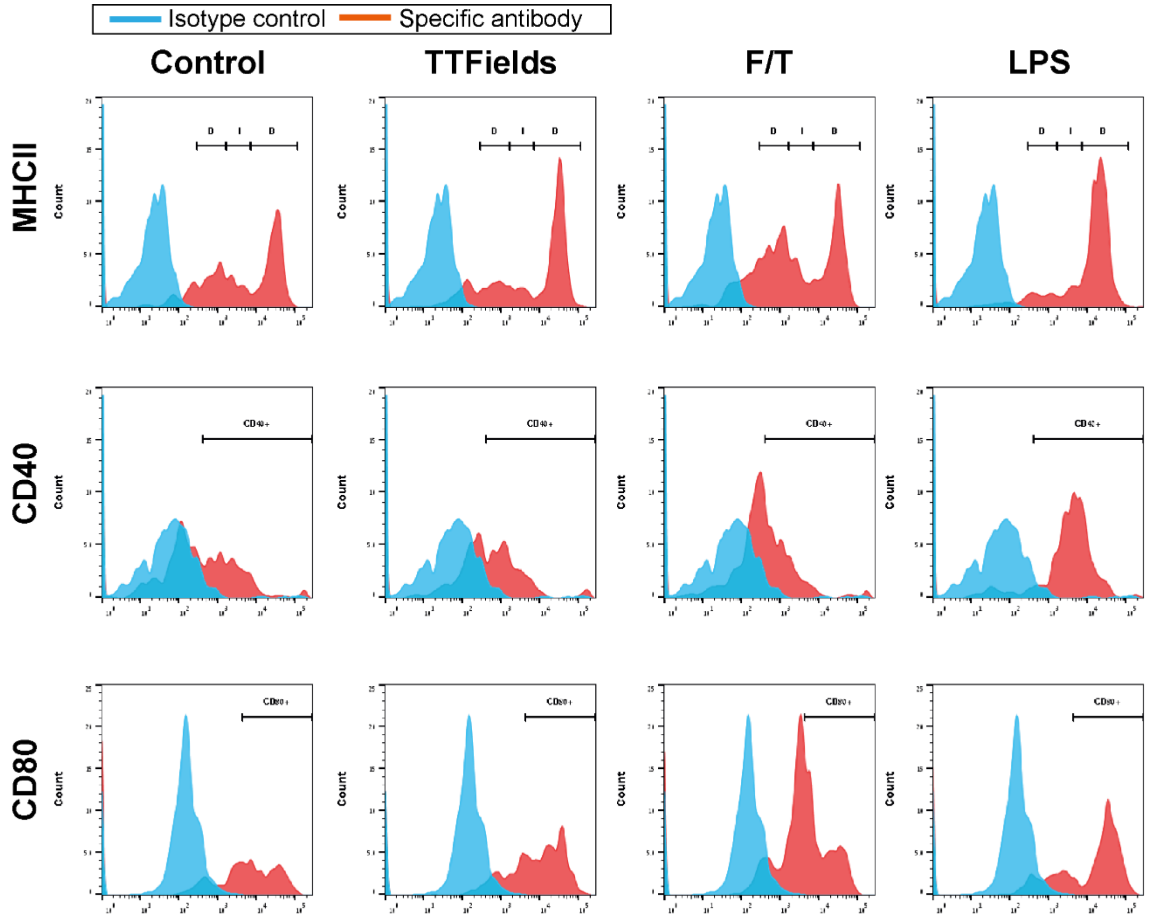


**Supplementary Figure 4. Culture of TTFields treated cancer cell suspension induces maturation of bone marrow derived dendritic cells.** Co-culture of untreated or TTFields treated, F/T treated or LPS treated LLC-1 cells and BMDCs. BMDCs maturation phenotype was assessed using flow cytometry. Representative FACS histograms (D-dim, I-intermediate, and B-bright expression).

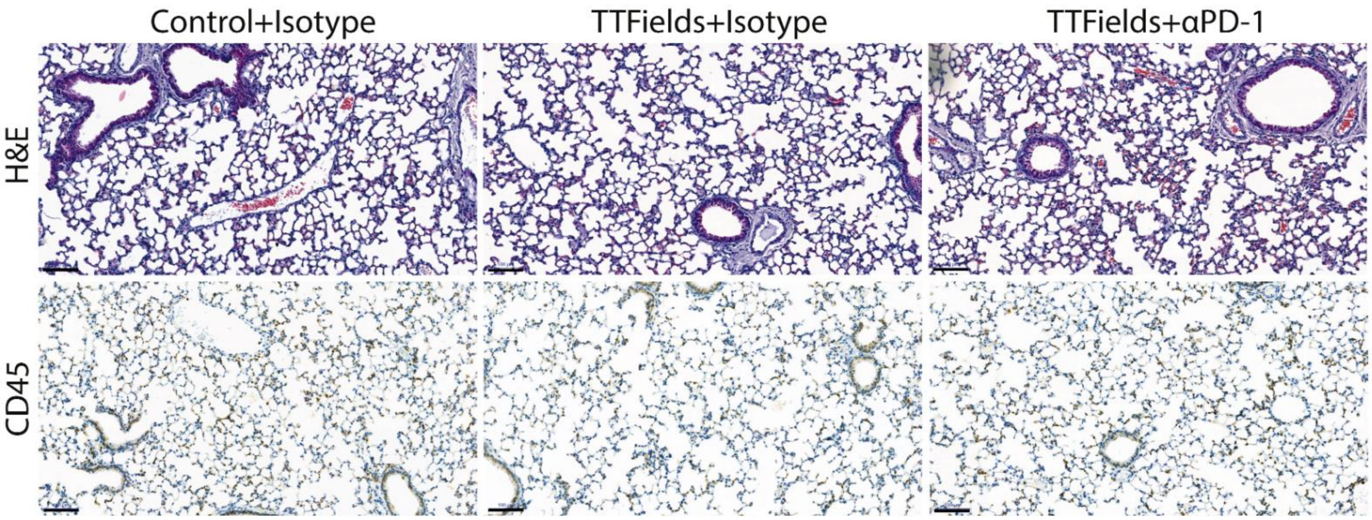


**Supplementary Figure 5. Application of TTFields to normal lung tissue.** Non–tumor-bearing C57Bl/6 mice (n = 3 mice per group) were treated with either sham heat transducers, TTFields, or TTFields in combination with anti-PD-1 therapy using the treatment schedule described in Fig 6A. Paraffin embedded sections (4μm thick) of the lungs from the different treatment groups were prepared and stained for evaluation of lung morphology (H&E; upper panel) and immune cell infiltrate (CD45; lower panel). Histopathological analysis of the section was performed by a certified veterinary pathologist (PATHO-LOGICA Ltd.) Scale bar=100μm.

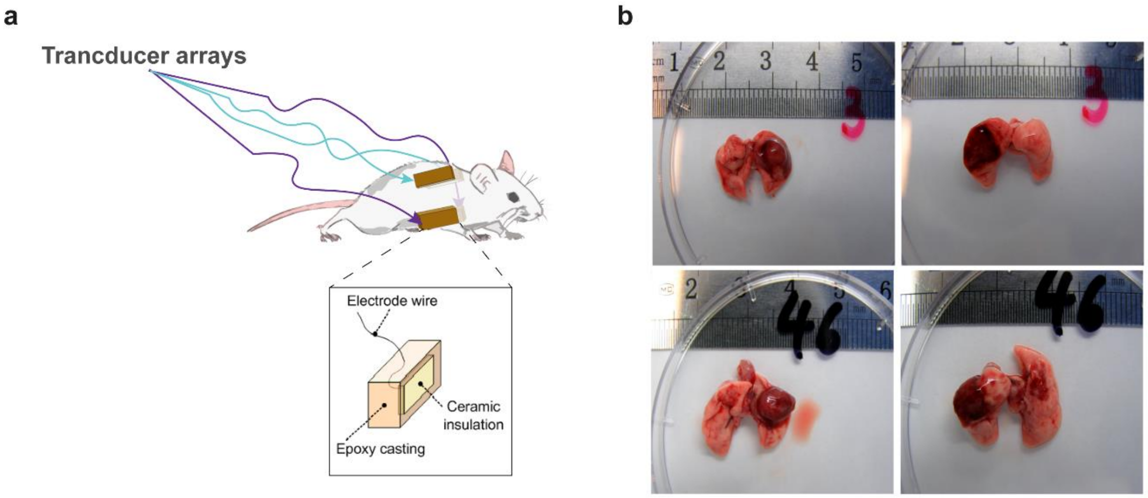


**Supplementary Figure 6. In vivo application of TTFields to the mouse thorax. (a)** For in vivo application of TTFields, a set of four insulated electrodes was placed around the mouse thorax so as to generate fields of two perpendicular directions. Control mice were treated by means of sham heat electrodes which were geometrically matched to the TTFields group. Each mouse was placed inside a separate cage and the electrodes were connected to the NovoTTF-100A device or the sham device. **(b)** Representative images of mouse lungs bearing a single LLC-1 tumor at day 15 following orthotopic implantation as described in the materials and methods.

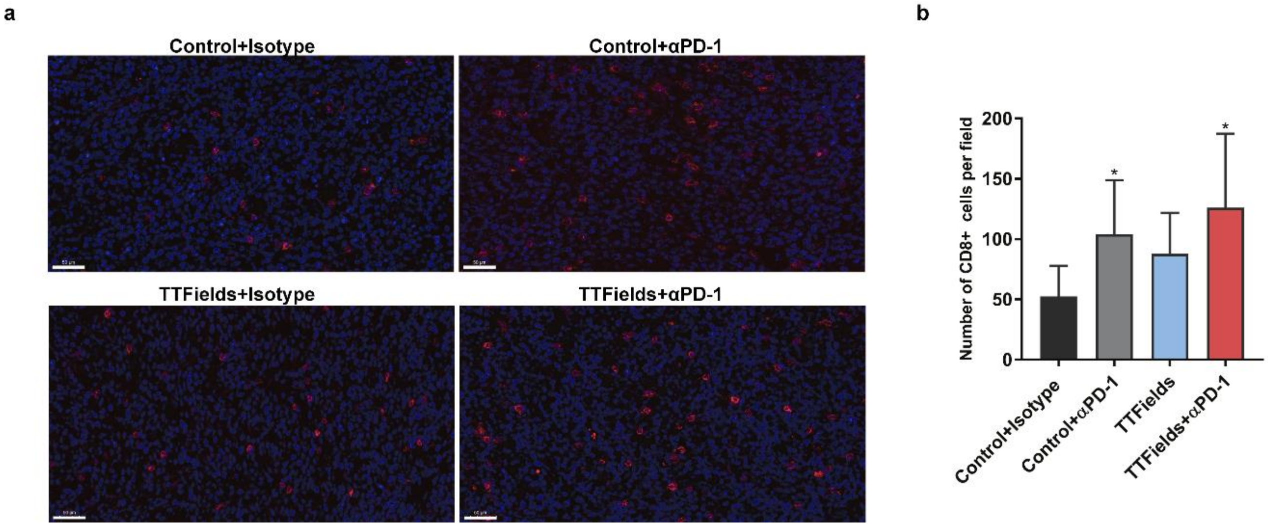


**Supplementary Figure 7. Relative CD-8 T cells infiltration in CT-26 tumors after treatment with TTFields or TTFields in combination with either αPD-1 or Isotype control.** **(a)** Ten-week-old female Balb/c mice bearing 60mm3 subcutaneous CT-26 tumors were treated with TTFields for 14 days, with a three days-break (days 13-16). Mice received an I.P. injection of anti-PD-1 (αPD-1) or Rat IgG2a. **(a)** Tumor CD8 immunostaining as a marker for cytotoxic T-cells infiltration (Red-CD8, Blue-DAPI; scale bar = 50 µm). **(b)** Quantification of **(a)**. Results are reported as mean ±SD of 5-6 measured fields per tumor with total of 5 mice per group. p values were determined using unpaired two-tailed t test for. *P < 0.05 from Control+Isotype group.

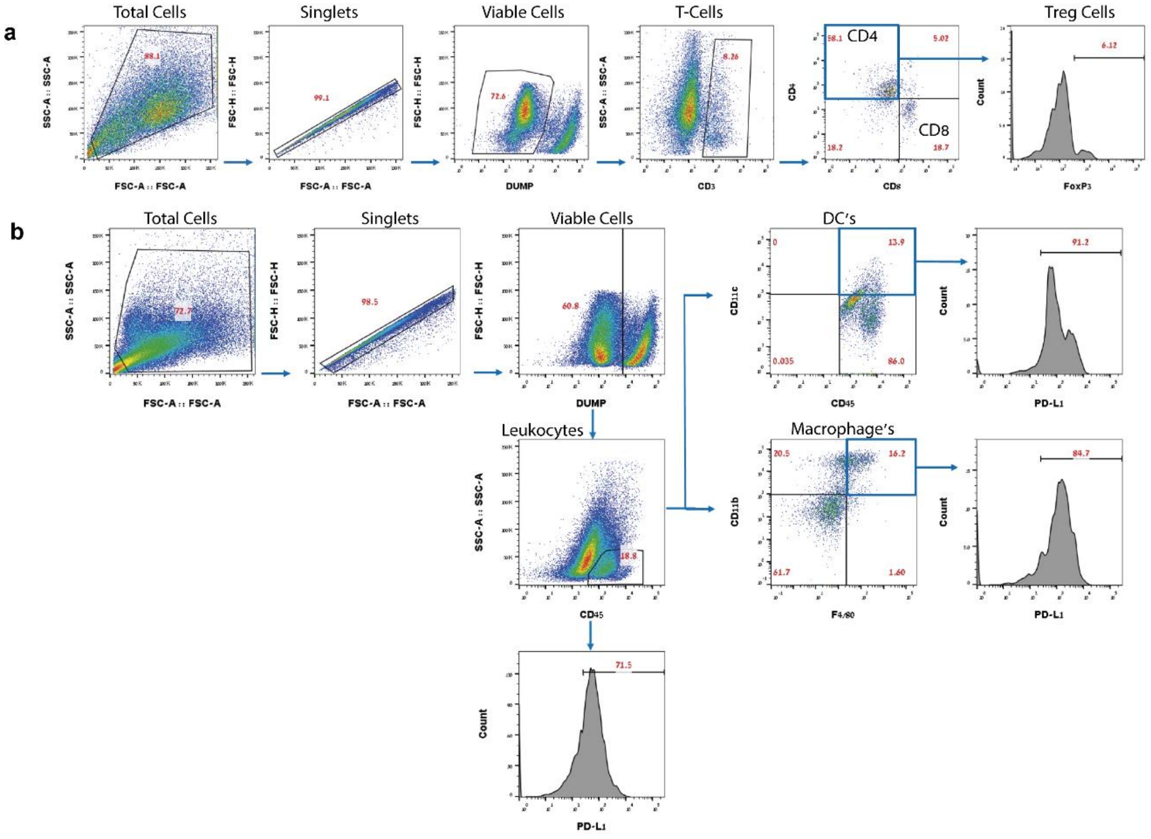


**Supplementary Figure 8. Flow cytometric gating strategy for discerning leukocyte subsets. (a)** Shown is a representative tumor analysis, whereby gated populations (from left to right) are indicated defining viable CD3+ T cells. These cells are differentiated into CD3+ CD4+ and CD3+ CD8+ populations. CD3+ CD4+ population is further gated on the basis of FoxP3 expression with CD3+ CD4+ FoxP3+ populations being associated with a T regulatory cells (Treg cells). **(b)** Shown is a representative tumor analysis, whereby leukocytes are gated based on CD45 expression. CD45+ cells are further divided into DCs and Macrophages subsets: DCs (CD45+CD11c+) and Macrophages (CD45+F4/80+CD11b+).All populations were further gated on the basis of PD-L1 expression.
